# Domestic Violence Against Women During the COVID-19 Pandemic and Its Relationship to Demographic and Family Factors: A Cross-Sectional Study in Iran

**DOI:** 10.7759/cureus.36633

**Published:** 2023-03-24

**Authors:** Farzan Khairkhah, Fatemeh Nasiri Amiri, Mostafa Javanian, Hossein-Ali Nikbakht, Mahbobeh Faramarzi, Jamileh Aqatabar Roudbari, Samih A Odhaib, Kayhane Mohammadi Aref, Hajar Habibpour

**Affiliations:** 1 Psychiatry, Social Determinants of Health Research Center, Babol University of Medical Sciences, Babol, IRN; 2 Reproductive Health, Social Determinants of Health Research Center, Babol University of Medical Sciences, Babol, IRN; 3 Infectious Disease, Infectious Diseases and Tropical Medicine Research Center, Health Research Institute, Babol University of Medical Sciences, Babol, IRN; 4 Biostatistics and Epidemiology, Social Determinants of Health Research Center, Babol University of Medical Sciences, Babol, IRN; 5 Psychology, Fatemeh Zahra Infertility and Reproductive Health Research Center, Babol University of Medical Sciences, Babol, IRN; 6 Public Health, Babol University of Medical Sciences, Babol, IRN; 7 Diabetes and Endocrinology, Faiha Specialized Diabetes, Endocrine and Metabolism Center, Basrah, IRQ; 8 Medicine, Babol University of Medical Sciences, Babol, IRN

**Keywords:** sociodemographic factors, physical violence, verbal abuse, coronavirus disease, domestic violence

## Abstract

Background: During the COVID-19 pandemic, we have witnessed increased complaints from third parties about violent conditions through social media. This study aimed to determine the prevalence of domestic violence (DV) against women following exposure to the COVID-19 pandemic and its relevance to some related factors.

Materials and methods: This study was conducted from July 2020 to May 2021 on married women of Babol, Iran. Eligible women entered into the study in a multi-stage cluster random sampling method. Data collection tools included demographic and family data, questionnaire HITS (Hurt, Insult, Threaten and Scream). Relationships were estimated using the Univariate and multivariate regression models.

Results: The mean age of 488 women and their spouses was 34.62 ± 9.14 and 38.74 ± 9.07, respectively. Of the total female participants, 37 (7.6%), 68 (13.9%), and 21 (4.3%) were victims of total violence, verbal abuse, and physical violence, respectively. Ninety-five women (19.5) had a history of coronavirus infection. Women who were satisfied with their income and husbands were university educated, their chances of DV were reduced by 72% (95% CI (0.09-0.85), OR = 0.28) and 67% (95% CI (0.11-0.92), OR = 0.33) respectively. Drug abuse by husbands increased the likelihood of DV by up to 4 times (OR = 4.00), and more contact with their husbands at home due to home quarantine was more than twice as likely to have DV (OR = 2.64).

Conclusion: Since the level of domestic violence was lower than before the coronavirus pandemic, it seems that most Iranian women were more under the support of their husbands during the coronavirus pandemic to endure the fear and panic caused by the pandemic. Women whose husbands had a university education and sufficient income had less domestic violence.

## Introduction

Domestic violence includes physical, sexual, psychological, and financial abuse, controlling actions, behaviors, or coercion. Domestic violence, often known as violence by a partner, affects women disproportionately. Of all three women, almost one of them was the victim of physical or verbal violence during their lifetime [1]. There is a legal definition of the domestic violence unit in different countries' jurisdictions. Also, there is significant variation around the world in terms of laws that protect victims of violence or whether there is special protection for those under the legal age [2]. The extent of domestic violence reporting varies in jurisdictions and cultures, but domestic violence is reported in all countries and socioeconomic groups, including socioeconomic variables [3]. The long-term effects of domestic violence are feelings of helplessness, lack of confidence, severe depression, physical injuries, and even more serious consequences like suicide [4].

After the WHO declaration recommended COVID-19 as an epidemic on March 11, 2020, social restricting practices were intended to decrease the spread of Infection worldwide. These measures have impacted family income, personal relationships, well-being, and dynamics [5]. Implementing social distancing and quarantine guidelines in early 2020 resulted in fewer community members and more time at home with family members. These measures were necessary to reduce the transmission of the disease; however, they were followed by fear, isolation, and economic and social pressures, causing experts to fear "a strong global increase in domestic violence" [6]. Since they restricted the trips to prevent the prevalence of COVID-19, there have been many concerns about DV in many countries, including China, France, Spain, Italy, and the UK [7]. Quarantine conditions compel women to spend more of their time in their home environments, and with the aggressor, the fact that makes both professional screenings and asking for help complicated [5]. During the COVID-19 pandemic, concerns have been expressed about the possibility of increased domestic violence in many countries [8]. Some researchers reported increasing complaints from third parties about violent situations via social media [9]. However, in Iran, no study has been conducted on this issue during the Coronavirus pandemic.

Several studies have examined the relationship between the outbreak of COVID-19 and its effect on domestic violence against women and have shown that the prevalence of COVID-19 caused symptoms including fear, loneliness, anxiety, anger, violence, severe depression, and post-traumatic stress [10-14]. Because of the COVID-19 pandemic, many people suffer from psychological-social problems, loss of jobs, and financial problems, which also likely cause domestic violence [15-18]. The hypothesis is that increasing family conflicts due to emotional, economic, and mental health challenges may lead to more DV. Spreading public awareness and public perception of the possibility of increasing the risk of DV in quarantine and the way we are engaged in support, screening, and intervention to address domestic, family, and partner violence, along with the implementation of public policies and focusing strategies on gender and rights, have been essential issues.

This study aimed to determine the prevalence of violence against women during the COVID-19 pandemic and its relationship to some demographic and familial factors.

## Materials and methods

Study design and participants

The cross-sectional study was conducted from July 2020 to May 2021 among married women in urban and rural communities in Babol, Mazandaran Province, Iran, to assess domestic violence against women and its relationship with some demographic factors, including age, spouse age, employment status, education, spouse education, income satisfaction, opioid drug use, Spouse opioid drug use, and familial factors including the number of children, Length of marriage in years, and home quarantine during the coronavirus pandemic including having less contact with family, death of relatives due to coronavirus virus, Infection of family members with coronavirus, history of infection coronavirus virus, health concerns during the coronavirus pandemic in Iran. 

The sample size was used for all the descriptive and analytical purposes of the study, and the largest sample size was used. For this purpose, using the sample size calculation formula to estimate the proportion in the population, taking into account the first type error, α = 0.05, and according to a similar study, P = 0.3 (19) and d = 0.05 were considered. Therefore, the required sample size for this study was determined to be 322 people. However, due to cluster sampling, the estimated number of samples was multiplied by the number of design effects = 1.5, based on which the minimum number was estimated to be 483 samples. However, because there was a possibility of incomplete completion of the questionnaire, initially, 527 women completed the questionnaires, and 7.4% of them were excluded from the study due to incomplete completion of the questionnaire. Finally, the statistical analysis was done on 488 women.

The sampling method was multi-stage sampling. For this type of sampling, Babol city was first divided into 4 regions (classes) based on geographical location, and then three health centers (as clusters) were randomly selected within each area. Within each healthcare center, samples were randomly selected from the list of eligible women according to the number of married women in that center and included in the study. A list of all married women in each center was prepared from the Sib system (Integrated health system), and each one of the selected samples was contacted by phone. According to the inclusion and exclusion criteria of the study, they were invited and written informed consent to participate in the research was obtained from eligible women in the relevant health centers.

The executive stages of the present study began after receiving the code of ethics from the ethics committee of Babol University of Medical Sciences (IR.MUBABOL.REC.1399.190) and receiving an introduction letter from the university and coordination with the authorities. First, all eligible individuals were invited, and written informed consent was obtained before participating in the study; their data would remain confidential, and their autonomy would be respected. 

The research community included families covered by the University of Medical Sciences health centers during the COVID-19 pandemic. In this case, the number of samples of each center and the base was prepared, and the total number of women was calculated; then, about the number of married women, samples were calculated according to the study's sample size for each center or base. Moreover, the samples were selected randomly from the women under the cover list.

Most selected samples were present to participate in the study, and selected samples unwilling to participate were replaced by the same random method. The selected women were then contacted and invited to participate in the study and were given a specific explanation and confidential information. When referring to the health center, they were given some explanation about the purpose of the study, optional participation in the study, the confidentiality of information in the study, and the possibility of knowing the study results. Then Conscious consent was obtained from the participants in written form, and data were collected using study tools.

Inclusion criteria of this study include willingness to participate in the study, families covered by the comprehensive health center, which have family cases, lack of chronic physical and mental illness, and married women who live with their husbands in the same house with or without children. Also, the exclusion criteria of this study include pregnant women, the incomplete completion of the questionnaire on the maximum effect of 5%, and mishaps, such as the death of relatives during the last three months.

Data collection tools

In explaining the objectives and methods of conducting the research, women were asked for the questionnaire completion, including demographic and familial information (age, education, the number of years of marriage, number of children, employment status, drug consumption, and alcohol consumption, income from the personal point of view) and spouses (age, career, education, drug consumption, smoking, and alcohol consumption) and COVID-19 pandemic condition (worrying due to outbreak of coronavirus, contact with family members, the death of the relatives due to the coronavirus and the infection issues) and screening questionnaire of violence, Hurts, Insults, Threaten and Screams (HITS). The HITS Screening Tool has proven helpful as a diagnostic tool. The tool includes four questions that physicians can provide to women via a questionnaire to assess the risk for Intimate Partner Violence (IPV). The short HITS questionnaire was designed by Sherin and colleagues (the family doctor in the US) to rapidly screen domestic violence [19]. This questionnaire focuses on 4 questions focusing on verbal and physical violence (hurt, insult, threaten and scream). Each question has five options based on the Likert scale with a minimum score of 1 to a maximum of 5, and the total score varies between 4 and 20. A score of more than 10 is considered domestic violence. Mirqafourian and colleagues in Iran have localized this questionnaire. To determine the validity of the HITS questionnaire, content, and formal validity were used, and Cronbach's alpha was 95%, and internal consistency ranges from 73 to 93 percent. The sensitivity and specificity of the HITS tool compared to the CTS2 questionnaire, a standard tool for identifying domestic violence, have been mentioned at 75.7 and 93.5, respectively [20].

Data analysis

Statistical analysis was carried out using SPSS version 20 Software. The descriptive characteristics of the patients with statistical markers for quantitative variables are presented by default normality using mean and standard deviation and the quality variables were expressed with frequency (relative frequency). To separate (the crude effects) and simultaneous (comparative effects), the predictor variables of violence (independent variables) were used with questions related to the condition of COVID disease (dependent variable), and logistic regressions were used to show their relationship. Since demographic characteristics and other factors can affect this relationship, these variables are considered in the final analysis to adjust if they were meaningful in univariate analysis. Variables significantly lower than 0.1 in univariate analysis were considered in the multivariate regression analysis. Multivariate binary logistic regression models reported the crude odds ratio (COR). For instance, some general characteristics were adjusted, and an adjusted odds ratio (AOR) and 95% confidence interval (95% CI) were reported. The statistical significance level was considered as p<0.05.

## Results

Among 527 participants, 7.4% were excluded from the study due to incomplete questionnaire completion, and statistical analysis was performed on 488 participants. The mean age of women and their spouses was 34.62 ± 9.14 and 38.74 ± 9.07, respectively. About half of the attendees had fewer than five years of marriage. Also, 171 (35%) women were employed, 202 (41.4%) had an academic education, 35(7.2%) were drug consumers, and 95 (19.5%) reported coronavirus infection. Of the total female participants, 37 (7.6%), 68 (13.9%), and 21 (4.3%) were victims of total violence, verbal abuse, and physical violence, respectively. Other demographic and family characteristics and COVID-19 Infection and domestic violence conditions during the COVID-19 pandemic were presented in table 1.

**Table 1:** Frequency and mean of demographic and social variables and the incidence of COVID-19 and domestic violence during the COVID-19 pandemic (n = 488).

Variable	Subgroup	SD±Mean Frequency (%)
Age	-	34.62 ± 9.14
Spouse age	-	38.74 ± 9.07
Number of children	-	1.40 ± 0.94
Number of years of marriage	<5	245 (50.20)
	≥5	195 (39.96)
	missed	48 (9.84)
Employment status	Unemployed	317 (65.0)
	Employed	171 (35.0)
Education	Up to 12 years	286 (58.6)
	University	202 (41.4)
Income satisfaction	No	349 (71.5)
	Yes	139 (28.5)
Spouse Education	Up to 12 years	314 (64.3)
	University	174 (35.7)
Opioid drug use	No	453 (92.8)
	Yes	35 (7.2)
Spouse opioid use	No	351 (71.9)
	Yes	137 (28.1)
Health concerns	I do not worry	41 (8.4)
	I'm a little worried	304 (62.3)
	I'm very worried	143 (29.3)
have less contact with family	Yes	407 (83.4)
	No	81 (16.6)
Death of relatives due to Coronavirus	Yes	48 (9.8)
	No	440 (90.2)
Infection of family members with Coronavirus	Yes	155 (31.8)
	No	333 (68.2)
Infection with the Coronavirus	Yes	95 (19.5)
	No	393 (80.5)
General violence	Yes	37 (7.6)
	No	451 (92.4)
verbal violence	Yes	68 (13.9)
	No	420 (86.1)
physical violence	Yes	21 (4.3)
	No	467 (95.7)

This study first investigated the correlation between the overall violence score with demographic and family characteristics and disease infection of COVID-19. For better results, verbal abuse and physical violence were measured by the study variables. Results of the regression model in examining the relationship between variables in the study with domestic violence at the time of the COVID-19 pandemic were shown in univariate analysis. Income satisfaction variables, education of spouse, drug use, and more spouse presence at home due to coronavirus quarantine with a total score of domestic violence had statistically significant relationships. So that people who were satisfied with their earnings worked as a prevention variable, and the chance of domestic violence in them was %72 (OR= 0.28) less than those who were not satisfied with their income. Also, domestic violence in women whose husbands had a university education decreased by 67% (OR=0.33) compared to women whose husbands had a lower education. The use of drugs by the spouse (OR= 4.00) and women having more contact with their spouse (OR= 2.64) had much DV. Variables with a P value less than 0.1 in univariate analysis were included in multivariate analysis. The results of the formulas for these four variables were also stable in the regression multivariate analysis. Thus, these four variables are independent and strong predictor variables for DV (table 2).

**Table 2:** Relationship between demographic and social characteristics and the incidence of COVID-19 disease with domestic violence during the COVID-19 pandemic (n = 488) *crude odds ratio; **fully adjusted odds ratio, adjusted for Number of children, Income satisfaction, Husband education, Drug use in husband, Being at home with family. OR, odds ratio.

Variable	Subgroup	Unadjusted		Adjusted	
		^*^(95% CI) OR	P-value	^**^(95% CI) OR	P-value
Age	-	1.02 (0.98-1.06)	0.207		
Spouse age	-	1.01 (0.97-1.05)	0.520		
Number of children	-	1.39 (0.98-1.97)	0.062	1.30 (0.87-1.94)	0.199
Number of years of marriage	<5	Reference group			
	≥5	1.28 (0.63-2.58)	0.488		
Employment status	Unemployed	Reference group			
	Employed	1.14 (0.57-2.27)	0.711		
Education	Up to 12 years	Reference group			
	University	0.57 (0.27-1.19)	0.138		
Income satisfaction	No	Reference group			
	Yes	0.28 (0.09-0.81)	0.020	0.28 (0.09-0.85)	0.025
Spouse Education	Up to 12 years	Reference group			
	University	0.26 (0.10-0.68)	0.006	0.33 (0.11-0.92)	0.035
Opioid drug use	No	Reference group			
	Yes	1.64 (0.54-4.93)	0.377		
Spouse opioid use	No	Reference group			
	Yes	4.28 (2.15-8.54)	< 0.001	4.00 (1.95-8.18)	< 0.001
Health concerns	I do not worry	Reference group			
	I'm a little worried	0.82 (0.27-2.51)	0.740		
	I'm very worried	0.54 (0.15-1.92)	0.347		
have less contact with family	Yes	Reference group			
	No	2.30 (1.08-4.87)	0.029	2.64 (1.18-5.90)	0.017
Death of relatives due to Coronavirus	Yes	Reference group			
	No	1.28 (0.37-4.25)	0.714		
Infection of family members with Coronavirus	Yes	Reference group			
	No	1.75 (0.78-3.92)	0.173		
Infection with the Coronavirus	Yes	Reference group			
	No	1.59 (0.60-4.21)	0.345		

For other variables, the chances of domestic violence in college-educated women are less than in women with lower education (OR=0.57), employed women compared to homemakers (OR=1.14), were higher, and for the status of coronavirus pandemic variables such as; Not being infected with the coronavirus by herself or her family members, even though they increase the chance of domestic violence, (OR =1.59) and (OR= 1.75) respectively, but none of these relations are statistically significant (table 2).

Also, the relationship between verbal abuse and physical violence with variables in this study of results indicated that the satisfaction of income and drug use by the spouse with verbal violence (Table 3) and the use of drugs by the spouse also with physical violence (Table 4) are statistically related.

**Table 3:** Relationship between demographic and social characteristics and the incidence of COVID-19 disease with verbal domestic violence during the COVID-19 pandemic (n = 488) *crude odds ratio; **fully adjusted odds ratio, adjusted for Income satisfaction, Drug use in husband. OR: odds ratio.

Variable	Subgroup	Unadjusted		Adjusted	
		^*^(95% CI) OR	P-value	^**^(95% CI) OR	P-value
Age	-	1.02 (0.99-1.05)	0.142		
Spouse age	-	1.01 (0.98-1.04)	0.349		
Number of children	-	1.16 (0.89-1.52)	0.262		
Number of years of marriage	<5	Reference group			
	≥5	1.26 (0.74-2.13)	0.388		
Employment status	Unemployed	Reference group			
	Employed	1.26 (0.74-2.13)	0.385		
Education	Up to 12 years	Reference group			
	University	0.85 (0.50-1.45)	0.569		
Income satisfaction	No	Reference group			
	Yes	0.29 (0.13-0.63)	0.002	0.30 (0.14-0.66)	0.003
Spouse Education	Up to 12 years	Reference group			
	University	0.66 (0.37-1.16)	0.154		
Opioid drug use	No	Reference group			
	Yes	1.60 (0.67-3.83)	0.286		
Spouse opioid use	No	Reference group			
	Yes	1.99 (1.17-3.39)	0.011	1.87 (1.09-3.21)	0.021
Health concerns	I do not worry	Reference group			
	I'm a little worried	1.64 (0.56-4.84)	0.363		
	I'm very worried	1.33 (0.42-4.18)	0.623		
have less contact with family	Yes	Reference group			
	No	1.68 (0.90-3.12)	0.101		
Death of relatives due to Coronavirus	Yes	Reference group			
	No	1.43 (0.54-3.76)	0.461		
Infection of family members with Coronavirus	Yes	Reference group			
	No	0.771 (0.45-1.31)	0.340		
Infection with the Coronavirus	Yes	Reference group			
	No	1.47 (0.72-2.99)	0.288		

**Table 4:** Relationship between demographic and social characteristics and the incidence of Covid-19 disease with physical domestic violence during the Covid-19 pandemic (n = 488) *crude odds ratio; **fully adjusted odds ratio, adjusted for Drug use in the husband, Corona in the family. OR: odds ratio.

Variable	Subgroup	Unadjusted		Adjusted	
		^*^(95% CI) OR	P-value	^**^(95% CI) OR	P-value
Age	-	1.02 (0.97-1.07)	0.311		
Spouse age	-	1.01 (0.96-1.06)	0.540		
Number of children	-	1.43 (0.91-2.25)	0.115		
Number of years of marriage	<5	Reference group			
	≥5	1.13 (0.45-2.85)	0.785		
Employment status	Unemployed	Reference group			
	Employed	1.14 (0.46-2.82)	0.764		
Education	Up to 12 years	Reference group			
	University	0.42 (0.15-1.18)	0.104		
Income satisfaction	No	Reference group			
	Yes	0.57 (0.19-1.75)	0.333		
Spouse Education	Up to 12 years	Reference group			
	University	0.41 (0.13-1.24)	0.115		
Opioid drug use	No	Reference group			
	Yes	1.38 (0.30-6.20)	0.671		
Spouse opioid use	No	Reference group			
	Yes	2.43 (1.01-5.86)	0.048	2.46 (1.01-5.95)	0.046
Health concerns	I do not worry	Reference group			
	I'm a little worried	2.22 (0.28-17.21)	0.445		
	I'm very worried	1.15 (0.12-10.59)	0.901		
have less contact with family	Yes	Reference group			
	No	1.19 (0.39-3.63)	0.758		
Death of relatives due to Coronavirus	Yes	Reference group			
	No	2.23 (0.29-17.05)	0.437		
Infection of family members with Coronavirus	Yes	Reference group			
	No	2.89 (0.84-9.98)	0.092	2.93 (0.84-10.13)	0.089
Infection with the Coronavirus	Yes	Reference group			
	No	1.47 (0.42-5.10)	0.542		

## Discussion

The study of domestic violence cases in Iran after the coronavirus pandemic was first carried out using a short questionnaire of HITS, which had 7.6%, 13.9%, and 4.3% victims of total violence, verbal abuse, and physical violence, respectively. The amount of domestic violence is similar to the study of Zheng et al. This study was conducted in Hong Kong, China, among pregnant women, and 15.62% of them were victims of violence. The participants had 90 cases of mental violence (11.07 %), 8 cases of physical violence (0.98 %), 7 cases of sexual violence (0.86 %), and 25 cases of (3.08%) having experienced all the above [21]. However, a study in Zimbabwe demonstrated a cross-sectional study titled Domestic violence among married women of fertility age; 42.71% had experienced domestic violence [22]. Another study cited a cross-sectional study on victims of domestic violence and linked it to mental distress in the Yangon region of Myanmar. The prevalence of domestic violence among married women in their lifetime was 61.8%, and over the last 12 months, 51.2% [23]. 

The prevalence of domestic violence in our study was less than in other countries [9]. The possible reasons for the decreasing prevalence of violence in this study may be because people's understandings of domestic violence vary in different regions and the fact that some women may suffer from violence, and they still do not take it as violence. Nevertheless, the same behavior in society and other cultures may be recorded as domestic violence. In Eastern culture, women are usually silent on preserving family stability and avoiding more violence, which may reduce domestic violence rates in Iran and China [24]. In addition to the difference between the prevalence of domestic violence against women in Iranian and international studies, there are differences in the reported practice, even in Iran. The reported prevalence in this study is less than the other Iranian studies done before the coronavirus pandemic [25]. Perhaps the pandemic has reduced domestic violence because people see themselves as kinder to each other, and the global conflict has brought people together.

Several factors can contribute to domestic violence. The present study showed that one of the predictors of domestic violence during the coronavirus pandemic is more presence of the spouse at home. The study's findings showed that women with more contact with their husbands at home due to home quarantine were more than twice as likely to have domestic violence.

According to Fegert et al., the long-term presence of spouses at home during the outbreak of COVID-19 disease caused opens up unresolved problems and increased sensitivity to defects and minor defects in marital relationships [26]. According to Sharma et al., there is also a connection between more presence of spouses at home and domestic violence [27]. From the viewpoint of Rauhaus et al., the recommendation to stay home has led to the emergence of domestic violence worldwide [28].

In addition to quarantine, the virus has increased economic consequences, including closing jobs and cutting down income, leading to domestic violence. With the outbreak of the COVID-19 virus, countries are forced to create necessary limitations in certain aspects of life; such a strategy has resulted in expulsion, loss of jobs, and reduced revenue. The small income is believed to lead to a rise in domestic violence. Even If a family does not have a history of violence, the problems of the economy during the pandemic due to financial pressures and lack of social support can fuel the violence [29]. The present study showed that income satisfaction is associated with a reduced likelihood of domestic violence. According to Bonomi and colleagues, the highest rate of domestic violence is related to poor areas [3].

Our study showed a direct relationship between spouse drug use and income dissatisfaction with domestic violence. Silverio et al. (2020) also reported that alcohol consumption and reduction of family income were among the factors contributing to the increase in domestic violence [30]. Our study had no significant relationship between female age and domestic violence.

Kaukinen et al. considered young women and economic dependence, alcohol, and drug abuse, and long-term unemployment contributing to increasing domestic violence. The effect of age variables is inconsistent with our study, which can be attributed to a different society [29]. In this study, no relation is observed between working or not working women with domestic violence. In the study of Lasong et al., the risk of domestic violence among working women was higher than among unemployed women [22], which is inconsistent with our study due to different communities and cultures.

One of this study's strengths was that the sampling technique was observed, but each study has limitations. One limitation of this study was that it was cross-sectional, so there are limitations in identifying causal relationships. Second, we cannot deny the reporting bias because of the self-reporting design of violence. Finally, there are limitations in the short form of HITS violence. Because this scale fails to present the severity of domestic violence, we should focus on women's empowerment and enable them to protect themselves and have better control over their lives. Therefore, it is suggested that all healthcare workers and health professionals have the necessary preparation to deal with different aspects of domestic violence and use the short domestic violence questionnaire for all clients as much as possible. If necessary, use consultations and practical strategies to reduce the risk of exposure and prevent domestic violence through timely intervention.

## Conclusions

Since the level of domestic violence was lower than before the coronavirus pandemic, it seems that most Iranian women were more under the support of their husbands during the coronavirus pandemic to endure the fear and panic caused by the pandemic. Women whose husbands had a university education and sufficient income were preventive variables for domestic violence. Drug use by the spouse and more contact with the spouse at home due to home quarantine increases the probability of domestic violence. 
